# Model-Driven Performance Optimization of Lead-Free RbGeI_3_ Perovskite Solar Cells Using SCAPS-1D

**DOI:** 10.1021/acsomega.6c04307

**Published:** 2026-08-08

**Authors:** Hariharan Rajasekaran, Thangaraji Vasudevan, Lung-Chien Chen

**Affiliations:** Department of Electro-Optical Engineering, 34877 National Taipei University of Technology, Taipei City 10608, Taiwan (ROC)

## Abstract

Driven by the urgent global requirement for sustainable and nontoxic photovoltaic materials, employing a combined DFT and SCAPS-1D computational framework, this study evaluates the electronic structure and photovoltaic performance boundaries, defined here as the efficiency ceiling set by intrinsic material quality at low defect density and the degradation floor imposed by high defect density and elevated operating temperature of RbGeI_3_-based solar cell architectures. The computational analysis identifies a maximum achievable efficiency of 31.75% under ambient thermal conditions (300 K), supported by first-principles validation of intrinsic material quality, identifying a critical performance threshold at defect densities near 1 × 10^14^ cm^–3^. By isolating the specific impact of interface trap states at both the HTL/perovskite and ETL/perovskite junctions, we demonstrate that RbGeI_3_ possesses significant intrinsic defect tolerance, maintaining performance stability even as defect levels approach 1 × 10^16^ cm^–3^. In this regime, key metrics such as the fill factor (FF) and open-circuit voltage (*V*_oc_) remain remarkably resilient against structural imperfections, whereas higher densities trigger enhanced nonradiative recombination that significantly degrades output. Furthermore, a broad-spectrum thermal analysis spanning 300–500 K provides a realistic roadmap for operational stability, showing that the device retains a high efficiency of 23.67% even under extreme heat. The results indicate that performance degradation at elevated temperatures is primarily driven by accelerated carrier recombination and reduced *V*_oc_ values. Ultimately, these findings highlight RbGeI_3_ as a robust, eco-friendly candidate for the next generation of high-efficiency, lead-free photovoltaics.

## Graphical abstract

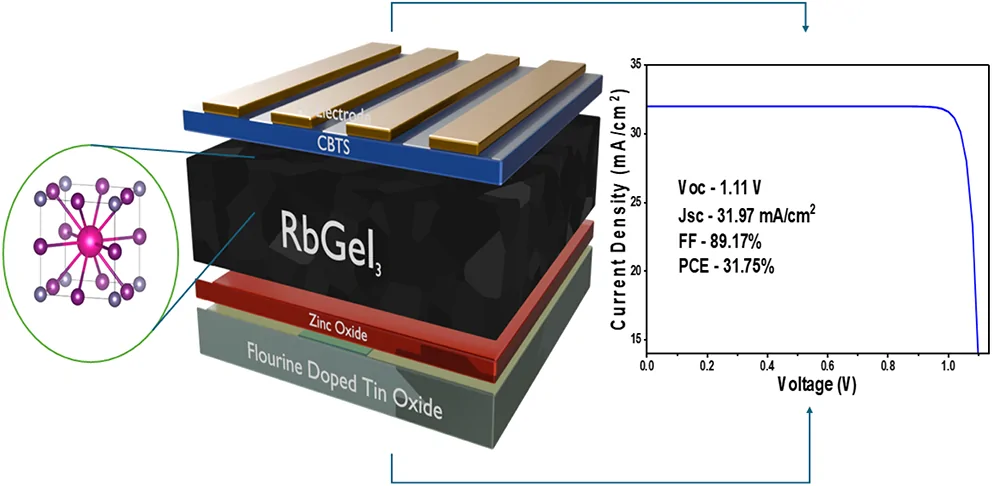

## 1 Introduction

The photovoltaic research landscape has experienced a dramatic transformation through halide perovskite development, witnessing efficiency progressions from initial proof-of-concept demonstrations to certified performance exceeding 25% within a single decade.^1–4^ This unprecedented advancement stems from favorable carrier transport properties, solution-phase fabrication compatibility, and compositional bandgap engineering capabilities. Nevertheless, lead incorporation in state-of-the-art compositions introduces multifaceted challenges encompassing environmental persistence, biological toxicity, and increasingly stringent regulatory frameworks.^5^ These constraints have catalyzed intensive efforts toward identifying nontoxic substitutes capable of maintaining the optoelectronic advantages characteristic of lead-based systems.^6,7^

Among lead-free alternatives, germanium (Ge^2+^)-based halide perovskites (of the form AGeX_3_) are particularly promising due to their potentially suitable bandgaps, good optical absorption, and favorable carrier transport when properly engineered.^8–10^ The combination of Sn and Ge (i.e., mixed Sn–Ge perovskites) has also been studied to balance stability and defect tolerance; in fact, reviews of Sn–Ge systems highlight the critical role of defect control and interface design in approaching high efficiency.^11–13^ Within the all-inorganic Ge perovskite family, RbGeI_3_ emerges as an attractive candidate: theoretical and computational studies of CDX_3_ perovskites (C = Cs, Rb, K; D = Ge, Sn) identify direct band gaps within the 1.1–2.0 eV interval and strong absorption coefficients, including for RbGeX_3_ systems, suggesting feasibility for effective light harvesting.^14–16^ Furthermore, Rb-based perovskites may stabilize lattice tolerance and reduce ionic disorder compared to larger A-site cations.^17,18^ Complementary computational and experimental studies of related lead-free absorber and buffer-layer chemistries further support the broader viability of this materials class for nontoxic photovoltaics.^19–22^ However, translating the intrinsic potential of RbGeI_3_ into high device performance requires careful interface engineering to suppress nonradiative recombination and ensure favorable band alignment at transport layers.^23–26^

Judicious engineering of both electron- and hole-selective contacts represents a critical optimization pathway, as junction-level defects or band discontinuities degrade *V*_oc_ and FF across all perovskite device categories, including lead-containing compositions.^27,28^ ZnO is a widely adopted n-type oxide ETL in perovskite photovoltaics, valued for its high electron mobility, wide band gap (transparent to visible light), and compatibility with low-temperature processing.^29–31^ Detailed reviews document the energy levels, trap states, processing methods, and stability issues of ZnO as a planar ETL in PSCs.^32,33^ However, ZnO surface basicity and its potential for catalyzing halide perovskite decomposition present challenges; interface passivation and buffer layers are commonly used to mitigate chemical instability. Staircase band alignment strategies (e.g., ZnO/Azol bilayers) have been used in perovskite systems to improve charge extraction and suppress recombination losses.^34–36^

On the side of hole transport, Cu_2_BaSnS_4_ (CBTS) is a promising inorganic candidate for HTL. CBTS offers favorable valence-band alignment, good hole mobility, and greater thermal/chemical robustness compared to many organic HTMs.^37–39^ In simulation studies, ETL/absorber/CBTS architectures (including ZnO + CBTS) have been predicted to produce high efficiency in Pb-free systems such as CsSnCl_3_ or double perovskites.^40–42^ For example, an SCAPS study showed that ZnO and CBTS used together can produce η ≥ 13% in Cs-based systems under optimized interface conditions.^43–45^ Furthermore, in a lead-free perovskite architecture, CBTS was evaluated and compared in combination with ZnO/Azolo ETLs. Because Ge perovskites are more sensitive to defects and interfacial recombination, a high-quality ETL/HTL pair with low trap densities and matched band offsets is essential to realize high *V*_oc_ and FF.^46–48^ In previous simulation works in RbGeI_3_, researchers have used SCAPS-1D to explore ETL and HTL combinations, optimize thicknesses and doping, and evaluate performance limits; however, experimental realizations with record efficiencies remain scarce.^49–52^ Addressing this shortcoming, we employ a two-tier computational strategy pairing ab initio electronic structure analysis with SCAPS-1D transport modeling, creating a direct link between quantum-mechanical ground-state properties and device-level performance predictions.

In this work, we present a fully inorganic RbGeI_3_ without lead solar cell architecture using a ZnO/RbGeI_3_/CBTS/Au device. Our optimized device achieves a PCE of 31.75%, *V*_oc_= 1.11 V, *J*_sc_ = 31.97 mA cm^–2^, and FF = 89.17% under AM 1.5G illumination. These metrics compete strongly with Pb-based perovskite records and surpass many theoretical projections for Ge-based systems.

## 2 Modeling of PSCs

### 2.1 Simulation Framework

We employed SCAPS-1D (University of Ghent)^53^ for all device modeling investigations. This one-dimensional solver addresses multilayer photovoltaic architectures by numerically resolving coupled semiconductor physics equations under a drift-diffusion approximation, enabling quantitative characterization of generation, recombination, and extraction phenomena. The framework proves particularly valuable for evaluating lead-free perovskite configurations, where defect distributions and heterojunction energetics critically govern achievable performance metrics.

### 2.2 Numerical Model and Governing Equations

Device performance evaluation employs the SCAPS-1D framework to solve three fundamental coupled transport equations governing carrier dynamics. Poisson’s equation correlates electrostatic potential gradients with spatial charge distributions originating from ionized dopants and mobile carriers. Charge conservation is maintained through electron and hole continuity relations, balancing optical generation against recombination while accounting for current density divergence. Transport physics incorporate field-driven drift and concentration gradient-driven diffusion components, with aggregate current representing combined electron and hole contributions.

Operating under steady-state conditions at a 300 K baseline temperature, the model assumes temporal invariance (∂*n*/∂*t* = 0) and one-dimensional transport geometry. Recombination pathways include Shockley–Read–Hall trap-mediated processes, band-to-band radiative transitions, and Auger multicarrier interactions. SRH mechanisms constitute the dominant nonradiative loss channel, particularly relevant to bulk and interfacial defect analysis central to this investigation. The solver achieves self-consistent solutions through iterative convergence with tolerance criteria of 10^–6^, incorporating wavelength-dependent optical generation profiles calculated from material absorption coefficients.

### 2.3 Boundary Conditions, Simulation Parameters, and Computational Procedure

Computational modeling proceeded under terrestrial solar spectrum conditions (AM 1.5G, 100 mW cm^–2^) with baseline thermal environment set at 300 K. Electrode interfaces employed ideal ohmic boundary conditions at both the front contact (FTO/ZnO electron collector) and rear contact (CBTS/Au hole collector). The computational procedure employed the SCAPS-1D solver under steady-state conditions, iteratively solving the fundamental semiconductor equations until self-consistency was achieved with a tolerance of 10^–6^, factoring in the optical generation profile calculated from layer absorption coefficients. A systematic parameter sweep was crucial for optimization, focusing on three key variables: bulk trap concentration, thermal operating conditions, and absorber geometry. The thermal analysis spanned 300 to 500 K, linking enhanced trap-assisted losses to efficiency reduction from 31.75% (300 K) to 23.67% (500 K). Similarly, the absorber bulk defect concentration was varied from a theoretical floor of 1 × 10^14^ cm^–3^ (yielding PCE = 33.8%) up to 1 × 10^20^ cm^–3^, with the practical operating point of PCE = 31.75% achieved at a low defect level, and a sharp decline to ∼1% observed at the highest concentrations due to enhanced trap-assisted recombination. From the final optimized current–voltage (*J*–*V*) characteristics, the core photovoltaic metrics were extracted (PCE = 31.75%, FF = 89.17%, *J*_sc_ = 31.97 mA cm^–2^, and *V*_oc_ = 1.11 V), supplemented by energy-band diagrams and recombination profiles for comprehensive mechanistic interpretation (Table 1).

**Table 1:** Materials and Electronic Parameters Defining the FTO/ZnO/RbGeI_3_/CBTS/Au Device Architecture Employed throughout Computational modeling^44,54,55^

Parameter	Unit	FTO	ZnO (ETL)	RbGeI_3_ (Absorber)	CBTS (HTL)
Thickness (*d*)	nm	500	40	500 (Optimal)	100
Bandgap (*E*_g_)	eV	3.5	3.3	1.31	1.9
Electron Affinity (χ)	eV	4	4.0	3.9	3.6
Relative Permittivity (ϵ_r_)	Unitless	9	9	23.01	5.4
Effective density of states (CB) (*N*_C_)	cm^–3^	2.2 × 10^18^	3.7 × 10^18^	2.8 × 10^19^	2.2 × 10^18^
Effective density of states (VB) (*N*_V_)	cm^–3^	1.8 × 10^19^	1.8 × 10^19^	1.4 × 10^19^	1.8 × 10^19^
Electron mobility (μ_n_)	cm^2^/(V s)	20	100	28.6	30
Hole mobility (μ_p_)	cm^2^/(V s)	10	25	27.3	10
Doping type	-	n-type	n-type	p-type	p-type
Doping concentration (*N*_D_/*N*_A_)	cm^–3^	*N*_D_ = 1 × 10^19^	*N*_D_ = 1 × 10^18^	*N*_A_ = 5 × 10^20^	*N*_A_ = 1 × 10^18^
Defect density (*N*_t_)	cm^–3^	1 × 10^15^	1 × 10^15^	Variable	1 × 10^15^

The electrical characteristics of the heterojunction interfaces specifically the ZnO/RbGeI_3_ (ETL/Absorber) and RbGeI_3_/CBTS (Absorber/HTL) junctions were modeled to account for interfacial recombination losses. These interfaces were characterized by their defect densities, capture cross-sections, and the energetic distribution of trap states, typically following a Gaussian distribution centered within the band gap. While both interfaces influence the overall charge extraction efficiency, the ETL/perovskite junction was identified as the primary site for potential losses, requiring a lower defect density to maintain high *V*_oc_ with the parameters detailed in Table 2. The simulation assumes that thermionic emission and tunneling mechanisms govern carrier transport across these boundaries. The Gaussian energetic distribution assumed for the interface trap states (Table 2) is the default and most widely used approximation in SCAPS-1D device modeling of perovskite heterojunctions; it is a modeling convenience rather than an experimentally measured distribution for RbGeI_3_ interfaces specifically, since no such experimental characterization currently exists for this lead-free system.

**Table 2:** Electronic Characteristics Governing Heterojunction Interfaces between the Absorber and Transport Layers within the Simulated Device Structure^a^

Interface parameter	Unit	ZnO/RbGeI_3_ (Front)	RbGeI_3_/CBTS (Back)
Defect type	-	a	a
Capture cross-section (electrons)	cm^2^	b	b
Capture cross-section (holes)	cm^2^	b	b
Energetic distribution	-	Gaussian	Gaussian
Energy level (*E*_t_)	eV	c	c
Characteristic energy	eV	d	d
Interface defect density (*N*_it_)	cm^–2^	e	e
Recombination model	-	SRH	SRH

a a—Neutral, b—1 × 10^–19^, c—0.6 (above *E*_v_), d—0.1, e—1 × 10^10^ to 1 × 10^15^.

### 2.4 First-Principles Electronic Structure Computations

Ab initio calculations utilized Quantum ESPRESSO with plane-wave basis sets under the density functional theory framework. The PBE GGA governed exchange-correlation interactions, while ultrasoft pseudopotentials described core electrons for Rb, Ge, and I species. The Pm3m cubic structure was relaxed until atomic forces dropped below 0.01 eV/Å (energy tolerance: 10^–6^ eV), using 50/500 Ry plane-wave cutoffs and a 4 × 4 × 4 k-point grid for self-consistent calculations. Postconvergence band structure mapping traversed critical zone boundaries (Γ–X–M−Γ–R–X|M) via non-self-consistent procedures, while density of states extraction leveraged a refined 12 × 12 × 12 k-grid with 0.02 Ry Gaussian broadening. Orbital-decomposed projections revealed atomic contributions to valence and conduction manifolds, with the Fermi level establishing the zero-energy reference. The raw PBE-GGA calculation yielded a direct gap of approximately 0.54 eV at the R point; consistent with the well-documented tendency of PBE-GGA to underestimate band gaps, a scissor correction was applied to obtain the derived bandgap (∼1.31 eV) used in this work, and this corrected value together with the lattice parameters was fed directly into SCAPS-1D transport simulations, bridging quantum-mechanical electronic structure with macroscopic device characteristics of RbGeI_3_ photovoltaics.

The PBE-GGA functional was selected because it offers a favorable balance between computational cost and reliable structural/mechanical predictions, and it is the exchange-correlation treatment most widely adopted in prior SCAPS-1D/DFT studies of RbGeI_3_ and related AGeX_3_ compositions, allowing direct comparison with the literature values used elsewhere in this work. We acknowledge that PBE-GGA is known to systematically underestimate absolute band gaps relative to hybrid functionals (e.g., HSE06) or GW-corrected calculations and that the present calculation also did not incorporate spin–orbit coupling (SOC), which is a comparatively smaller correction for Ge-based systems than for the heavier Pb-based analogues but is nevertheless non-negligible near the conduction band minimum. Consistent with this known underestimation, the raw PBE band structure gives a direct gap of approximately 0.54 eV at the R point of the Brillouin zone; a scissor correction was applied to bring this in line with the higher gap values expected for RbGeI_3_ from the broader literature, yielding the 1.31 eV value used to seed the SCAPS-1D absorber parameters.

This two-tier computational approach combining quantum-mechanical ground-state calculations with drift-diffusion device modeling ensures that material parameters feeding into SCAPS-1D carry first-principles justification rather than relying solely on literature-sourced values.

## 3 Results and Discussion

### 3.1 Energy Band Alignment and Device Structure

Before examining macroscopic device behavior, we first establish the quantum-mechanical foundation of RbGeI_3_ through DFT-derived electronic structure, ensuring that subsequent device simulation parameters are grounded in first-principles calculations. Figure 1a presents the architectural design implementing sequential heterojunction layers: FTO transparent conductor substrate, ZnO electron extractor (n-type, 40 nm), RbGeI_3_ photoactive region (p-type, 500 nm), CBTS hole extractor (p-type, 100 nm), and Au rear metallization. This planar n–i–p configuration establishes unidirectional carrier-selective extraction pathways. Upon illumination, photogenerated charge carriers within the RbGeI_3_ absorber undergo efficient separation at the heterointerfaces, with electrons preferentially transported toward the ZnO layer and holes extracted through the CBTS layer. The employment of RbGeI_3_ as a nontoxic absorber material, combined with inorganic ZnO and CBTS transport layers, ensures an environmentally benign device architecture while maintaining thermal robustness, strong light-harvesting capability across sunlight, and efficient carrier mobility characteristics.

**Figure 1:**
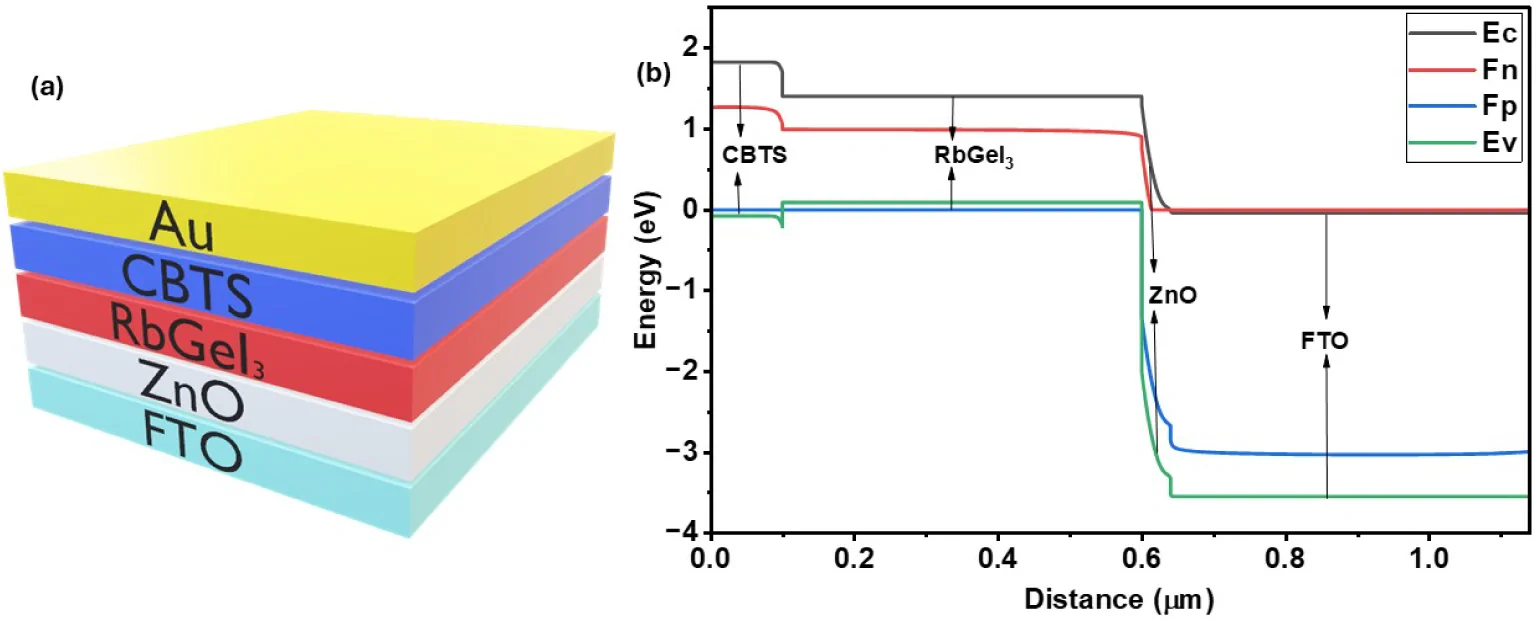
(a) 3D schematic of the simulated ZnO/RbGeI_3_/CBTS/Au perovskite solar cell. (b) Equilibrium energy band diagram showing *E*_c_, *E*_v_, and the electron and hole quasi-Fermi levels (*F*_n_, *F*_p_), indicating efficient interfacial charge transfer.

Figure 1b reveals an equilibrium band structure establishing substantial potential gradients throughout the RbGeI_3_ layer, providing a driving force for photogenerated carrier separation. Minimal conduction and valence band discontinuities at both heterojunctions (ZnO/RbGeI_3_ and RbGeI_3_/CBTS) suppress interfacial recombination while enabling a *V*_oc_ approaching 1.11 V. The absence of pronounced band-edge discontinuities (neither cliff-type nor spike-type barriers) at these critical junctions indicates excellent electronic compatibility between adjacent layers, representing a crucial factor in achieving the simulated high PCE approaching 31.75%. The electron and hole quasi-Fermi level gradient maintains near-ideal characteristics, reflecting minimal interfacial trap activity and facile charge separation. This junction architecture delivers balanced majority carrier extraction for both species while restricting recombination at the heterointerfaces. Such favorable band engineering provides the basis for achieved computational performance and confirms RbGeI_3_ suitability within environmentally benign, high-efficiency solar conversion frameworks.

### 3.2 Electronic Structure Analysis of RbGeI_3_

To understand the fundamental electronic properties of RbGeI_3_, density functional theory calculations were performed. Figure 2 presents the partial density of states (PDOS), revealing distinct orbital contributions from each constituent atom. The valence band maximum (VBM) is predominantly composed of I-p orbitals (peak ∼10 states/eV near 0 eV), with minor Ge-p and Ge-d contributions. This halide-dominated character facilitates efficient hole transport, crucial for minimizing recombination at the HTL interface. The conduction band minimum (CBM) exhibits strong Rb-p orbital character (peak ∼12 states/eV at −7 eV) along with significant I-s contributions (peak ∼7 states/eV at −8 eV). Ge-p orbitals contribute broadly across the conduction region, with peaks reaching 10 states/eV around 2 eV. The well-defined energy gap of approximately 1.31 eV, consistent with SCAPS-1D parameters, falls within the optimal range for single-junction photovoltaics according to the Shockley–Queisser limit.

**Figure 2:**
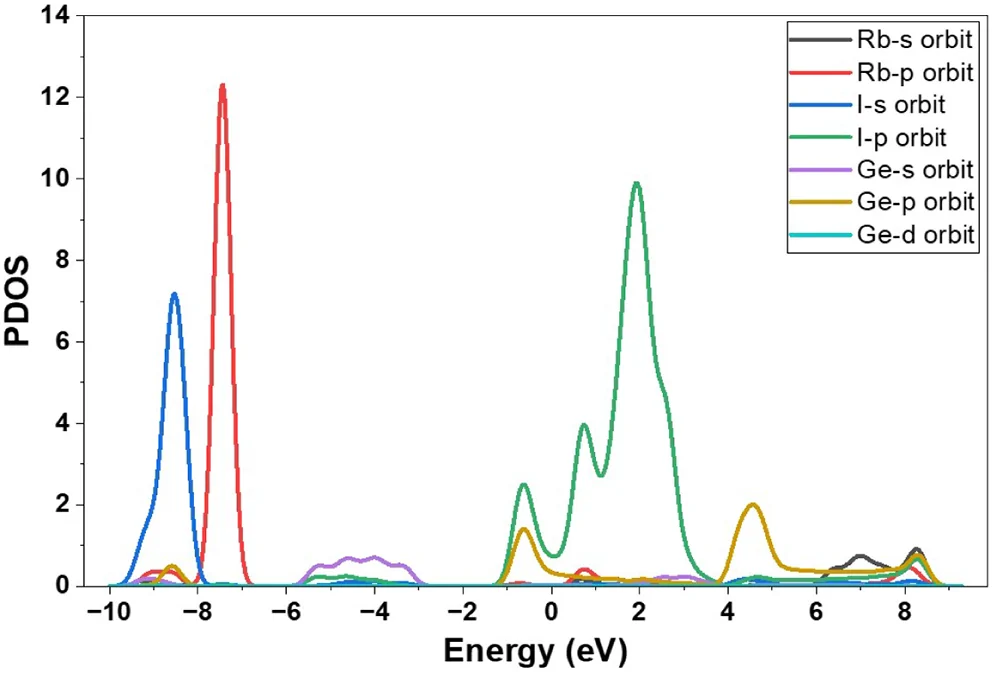
Partial density of states (PDOS) for RbGeI_3_ showing orbital-resolved contributions from Rb (s, p), I (s, p), and Ge (s, p, d) atoms.

Figure 3 displays the crystal structure and electronic band dispersion. Figure 3a shows the perovskite lattice arrangement with Ge atoms (pink, center) coordinated by I atoms (purple), forming the characteristic ABX_3_ structure with Rb cations occupying the A-site positions. Figure 3b presents the calculated band structure along high-symmetry points (Γ–X–M−Γ–R–X|M) in the Brillouin zone. Critically, at the R point in the baseline uncorrected band dispersion scheme, both the VBM and CBM occur. While the raw, uncorrected PBE-GGA treatment predictably yields a narrow direct gap of 0.54 eV at this high-symmetry point due to characteristic derivative discontinuities, the implementation of a rigid scissor shift correction establishes the effective operating bandgap parameter of 1.31 eV, utilized to seed the SCAPS-1D transport models. This direct gap enables efficient optical absorption without phonon assistance. The valence bands show moderate dispersion (∼5 eV bandwidth) with stronger curvature along Γ–X and Γ–M directions, indicating anisotropic hole transport. The conduction bands exhibit steeper dispersion, suggesting lighter electron effective masses and higher mobility (μ_n_ = 28.6 cm^2^/(V s) vs μ_p_ = 27.3 cm^2^/(V s)), beneficial for balanced ambipolar transport.

**Figure 3:**
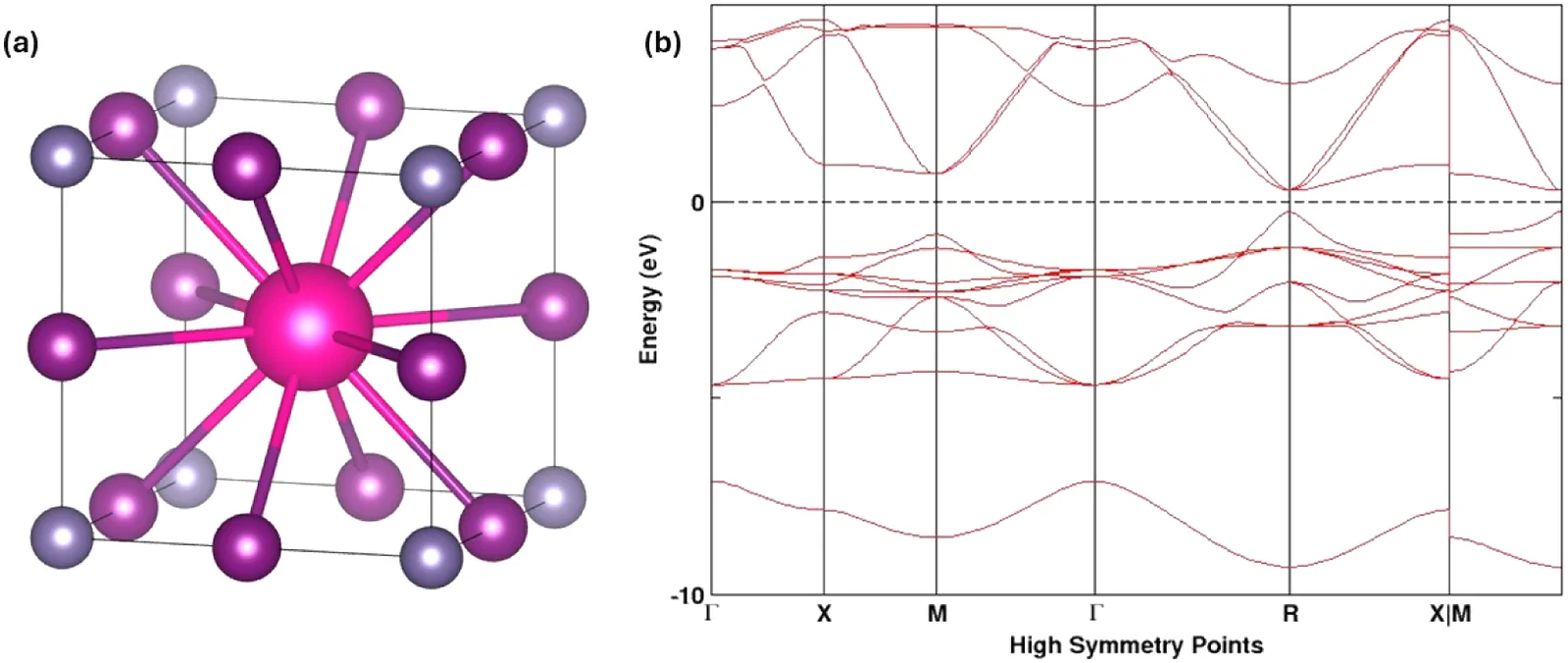
(a) Crystal structure of RbGeI_3_ perovskite showing the three-dimensional arrangement of atoms. (b) Electronic band structure calculated along high-symmetry directions, demonstrating direct bandgap behavior at the R point.

The absence of mid-gap states in the band structure confirms low intrinsic defect densities, supporting the baseline value of *N*_t_ = 1 × 10^14^ cm^–3^ used in simulations. The direct-gap nature, strong orbital hybridization at band edges, and favorable dispersion characteristics provide a quantum-mechanical foundation for the high PCE (31.75%) and fill factor (89.17%) achieved in device modeling. These DFT results validate RbGeI_3_ as an intrinsically promising nontoxic absorber material for next-generation photovoltaics.

### 3.3 Absorber Layer Thickness Optimization

Absorber geometry optimization involved RbGeI_3_ thickness parametric variation spanning 300 to 1000 nm (Figure 4). This parametric study revealed that changes in absorber thickness directly affect all major photovoltaic performance indicators through competing mechanisms of optical absorption enhancement and increased recombination losses. At the thinnest absorber configuration of 300 nm, the device produced an overall PCE of 28.71%, *J*_sc_ of 28.63 mA cm^–2^, FF of 89.24%, and *V*_oc_ of 1.12 V. The comparatively lower *J*_sc_ observed at this thickness stems from incomplete utilization of the incident sunlight, as the reduced optical path length limits photon absorption probability and consequently restricts photogenerated carrier production. Despite this limitation, the high FF and *V*_oc_ suggest minimal recombination losses due to short carrier transport distances.

**Figure 4:**
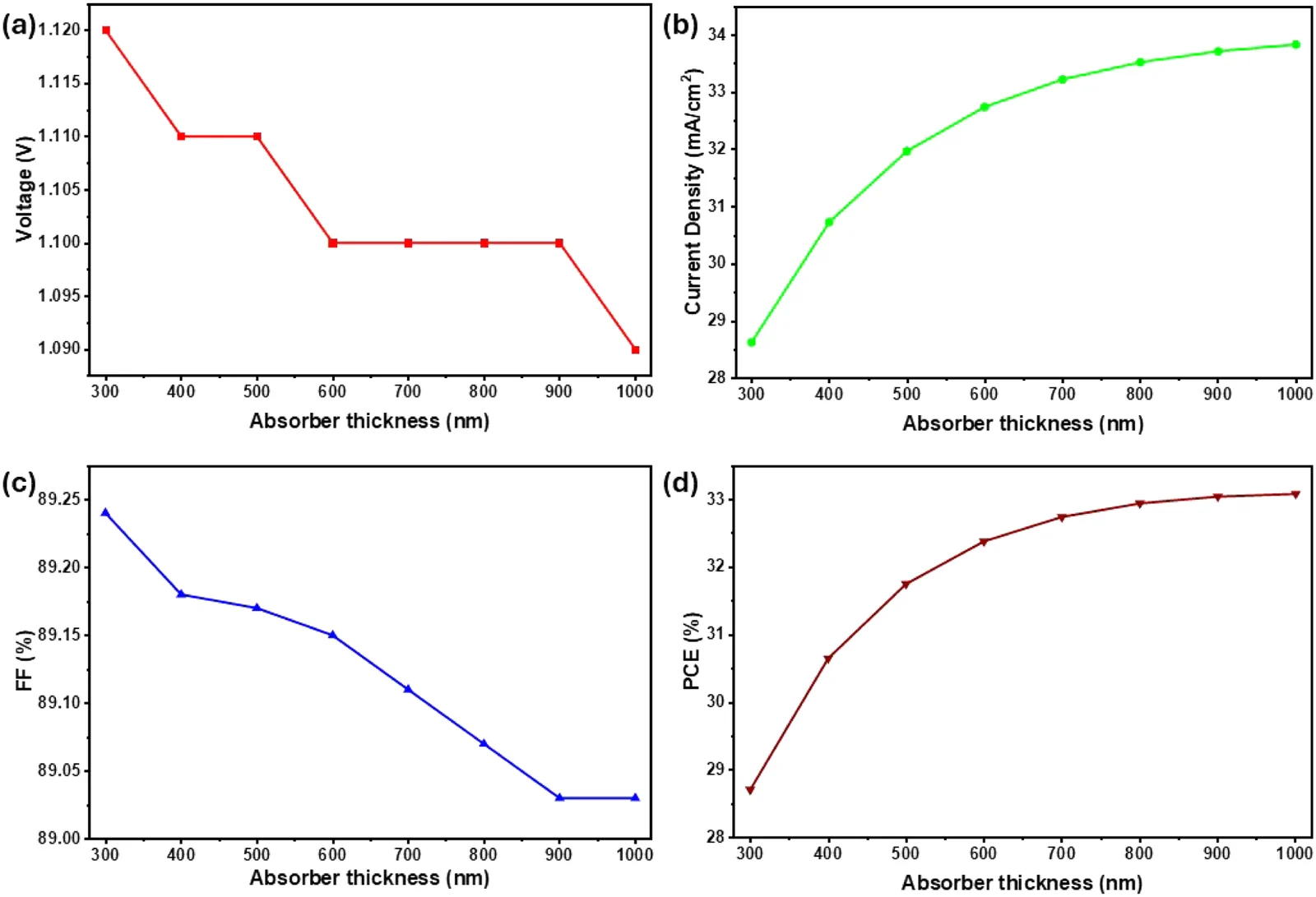
Effect of RbGeI_3_ absorber layer thickness on (a) *V*_oc_, (b) *J*_sc_, (c) FF, and (d) PCE.

Increasing the RbGeI_3_ thickness to 500 nm produced a substantial enhancement in photocurrent density, reaching 31.97 mA cm^–2^, while the overall efficiency improved to 31.75%. Notably, the *V*_oc_ and FF remained essentially constant at 1.11 V and 89.17%, respectively, indicating that this thickness increase enhances optical absorption without introducing significant additional recombination pathways or series resistance. This behavior suggests that the optimized thickness strikes an effective balance between photon harvesting efficiency and carrier collection probability. Absorber layers thicker than 500 nm showed diminishing returns, with *J*_sc_ reaching only 33.83 mA cm^–2^ at 1000 nm, while *V*_oc_ and FF deteriorated to 1.09 V and 89.03%, respectively. Enhanced recombination in thicker films increases the probability of charge loss during transport, offsetting absorption gains. Optimal performance occurs at 500 nm, effectively balancing light absorption and carrier extraction. Minimal improvement beyond 700 nm confirms that efficient photon capture occurs within the initial hundreds of nanometers, enabling material-efficient device designs.

### 3.4 Influence of Defect Density

The effect of trap density in the RbGeI_3_ absorber on device performance was examined through systematic variation spanning from 1 × 10^14^ to 1 × 10^20^ cm^–3^, with the resulting photovoltaic parameters compiled in Figure 5. This investigation reveals a pronounced correlation between defect density and device metrics, with performance exhibiting distinct behavioral regimes rather than simple linear degradation. The device simulated with the lowest trap density (1 × 10^14^ cm^–3^) yielded performance metrics of 33.82% (PCE), 89.16% (FF), 32.18 mA cm^–2^ (*J*_sc_), and 1.17 V (*V*_oc_). Extending simulations to lower defect concentrations (≤10^13^ cm^–3^) generated unrealistic efficiency predictions exceeding 36%, which go beyond the fundamental thermodynamic efficiency boundary defined by Shockley and Queisser for 1.31 eV single-junction devices. These nonphysical outcomes reflect idealized conditions implying perfect radiative recombination and complete carrier collection efficiency, which remain unattainable in practical thin-film materials. Consequently, 1 × 10^14^ cm^–3^ was adopted as the realistic lower boundary representing the highest crystalline quality achievable in optimized polycrystalline films without violating fundamental physical constraints.

**Figure 5:**
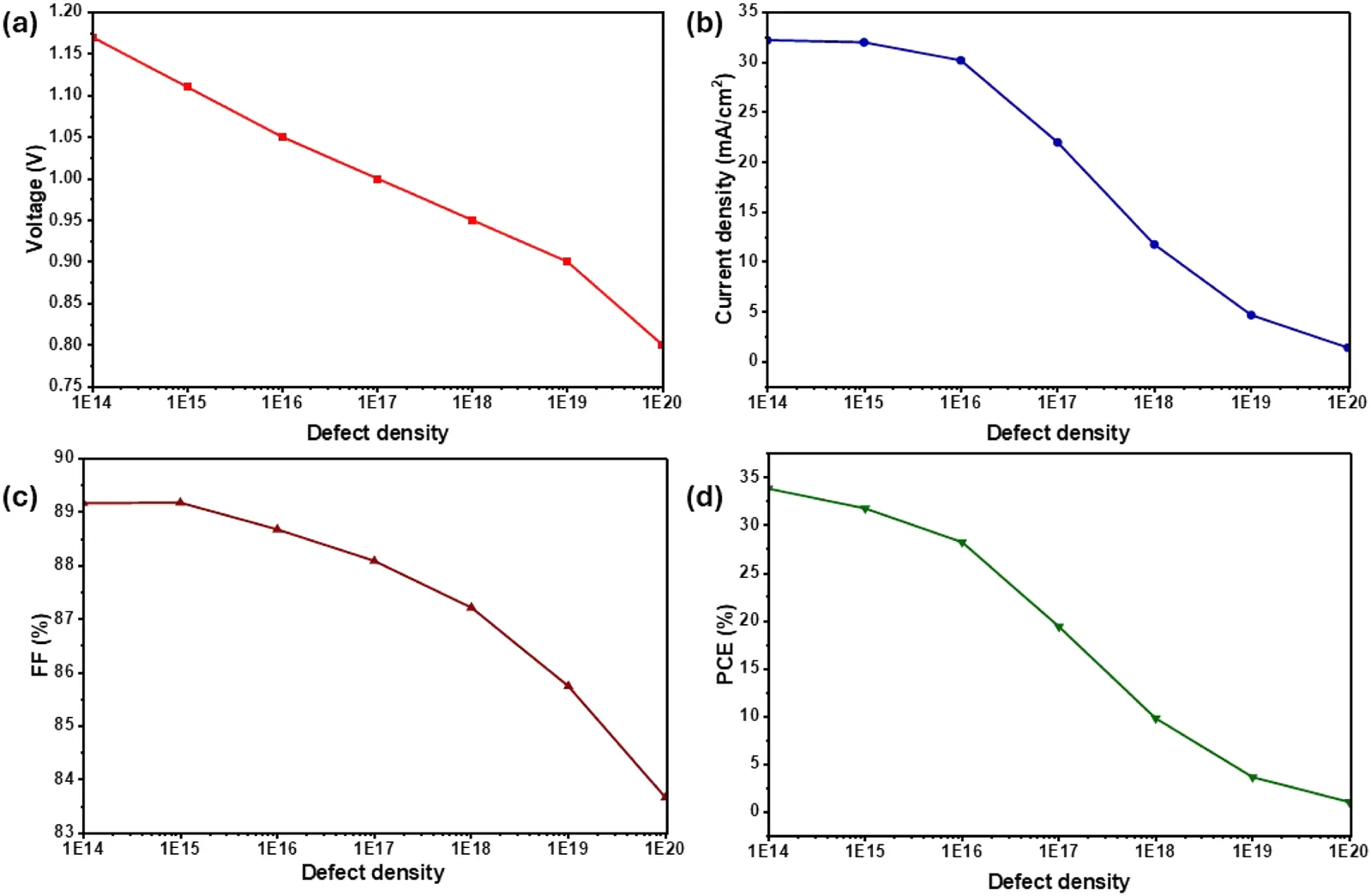
Variation of photovoltaic parameters (a) *V*_oc_, (b) *J*_sc_, (c) FF, and (d) PCE with absorber defect density.

Elevating the defect density to 1 × 10^15^ cm^–3^ induced a modest efficiency reduction to 31.75%, attributable primarily to minor *V*_oc_ losses, while *J*_sc_ and FF remained largely unaffected. This suggests that at moderate defect concentrations, radiative recombination continues to dominate over SRH processes. At 1 × 10^16^ cm^–3^, more substantial recombination effects emerged, manifesting as PCE = 28.22%, *J*_sc_ = 30.16 mA cm^–2^, and *V*_oc_ = 1.05 V, signaling the onset of trap-assisted recombination as a significant loss mechanism. When defect density surpassed 1 × 10^17^ cm^–3^, device performance deteriorated rapidly. At 1 × 10^18^ cm^–3^, efficiency collapsed to 9.77%, and at the highest examined concentration of 1 × 10^20^ cm^–3^, only approximately 1% efficiency persisted, indicating that trap-assisted recombination completely dominates charge transport dynamics at elevated defect levels.

The observed trends in *V*_oc_ and FF underscore the influence of SRH recombination mechanisms, which progressively reduce the diffusion length and carrier lifetime as trap state density increases. The reduction in FF from 89.16% to 83.66% across this defect range reflects the degradation of diode ideality due to defect-mediated leakage currents and increased series resistance effects. The precipitous *V*_oc_ decline from 1.17 to 0.8 V demonstrates how trap states positioned near midgap become increasingly effective recombination centers at higher densities, consequently reducing the achievable photovoltage and degrading the quasi-Fermi level divergence.

These findings establish that even moderate increases in bulk defect concentration can substantially compromise carrier collection efficiency and overall device performance. These findings highlight that preserving trap concentrations below 1 × 10^15^ cm^–3^ is essential for sustaining high device performance, to preserve the intrinsic electronic quality of RbGeI_3_ and achieve efficiencies approaching theoretical limits. This requirement translates to practical fabrication challenges, likely necessitating carefully controlled crystallization conditions, compositional optimization, or postdeposition passivation treatments to minimize deep trap state formation in experimental devices.

We emphasize that the claim of intrinsic defect tolerance up to *N*_t_ approaching 10^16^ cm^–3^ refers specifically to the comparatively mild roll-off in FF and *V*_oc_ observed in this intermediate regime (Section 3.4, Figure 5), not to defect immunity: PCE still falls from 33.82% at 10^14^ cm^–3^ to 28.22% at 10^16^ cm^–3^ and collapses beyond 10^17^ cm^–3^ as SRH recombination becomes dominant. This behavior is consistent with the shallow, halide-dominated trap character typically reported for iodide-rich Ge and Sn perovskites, in which trap levels lie closer to the band edges than to mid-gap, reducing their capture efficiency relative to deep midgap defects. On the fabrication side, achieving absorber defect densities below 10^15^ cm^–3^ realistically requires a combination of strategies reported for related lead-free perovskites: antisolvent-assisted or vacuum-flash crystallization to reduce grain-boundary trap density, stoichiometric excess or reducing additives (e.g., SnF_2_, hypophosphorous acid, or hydrazinium salts for Ge/Sn systems) to suppress halide vacancies and B-site oxidation, and postdeposition surface passivation with Lewis-base or ammonium-halide treatments to neutralize under-coordinated surface defects.

### 3.5 Temperature Dependence

We evaluated thermal stability by measuring performance metrics across temperatures ranging from 300 to 500 K (Figure 6). Our simulations demonstrate that while photocurrent generation (*J*_sc_) exhibits minimal temperature sensitivity, voltage-related parameters including *V*_oc_ and FF undergo systematic degradation with increasing thermal energy. At 300 K, the device shows optimum performance with PCE = 31.75%, FF = 89.17%, *J*_sc_ = 31.97 mA cm^–2^, and *V*_oc_ = 1.11 V. When the temperature rises to 350 K, the *V*_oc_ drops slightly to 1.06 V, and FF drops to 87.37%, reducing the PCE to 29.78%. Further temperature increase to 500 K causes a pronounced decline in *V*_oc_ (0.90 V) and FF (81.46%), resulting in a PCE of 23.67%.

**Figure 6:**
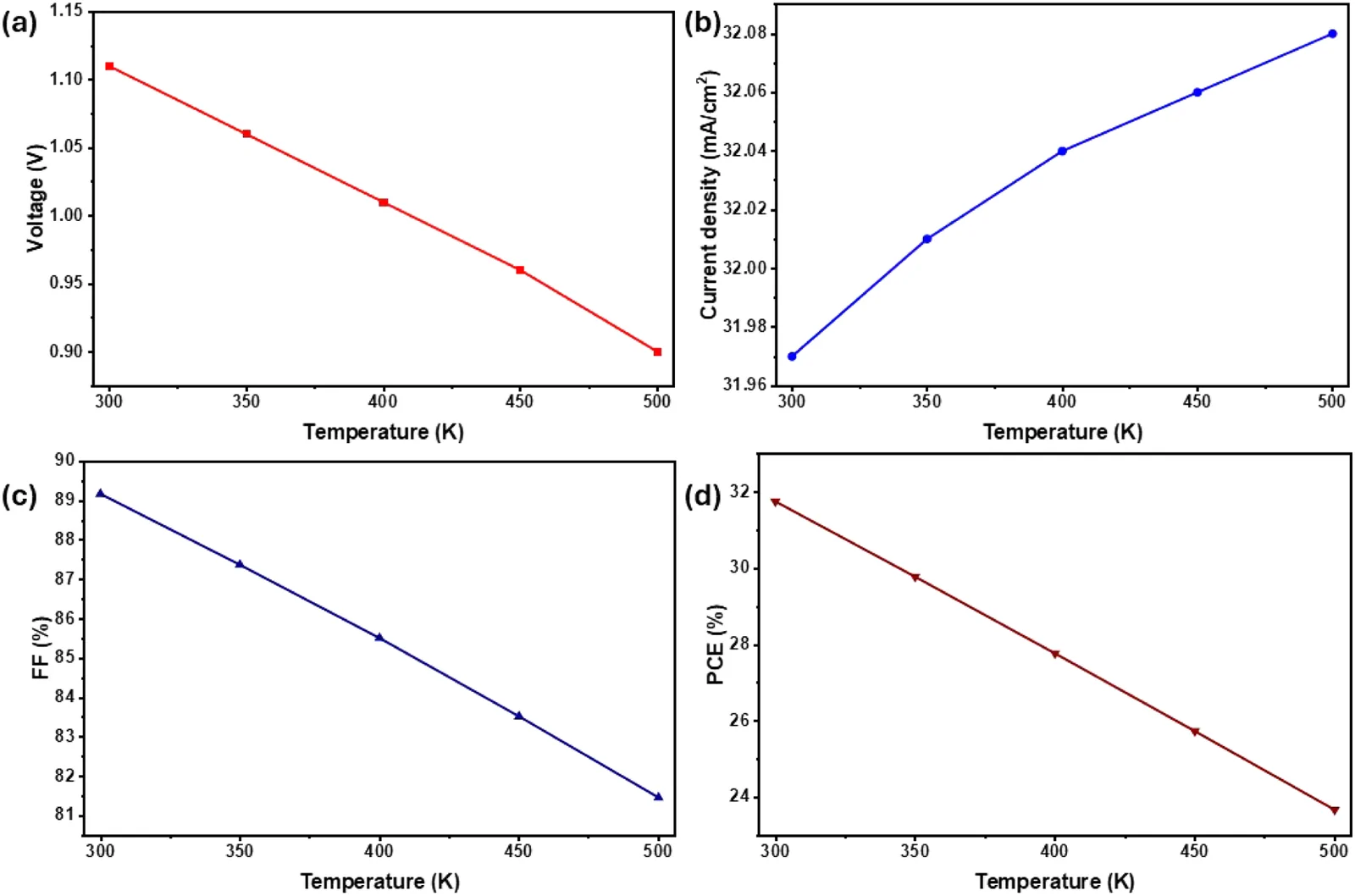
Variation of (a) *V*_oc_, (b) *J*_sc_, (c) FF, and (d) PCE with temperature (300–500 K).

Elevated operating temperatures drive systematic *V*_oc_ reduction through thermally activated intrinsic carrier proliferation, intensifying trap-mediated losses and weakening internal field gradients according to *V*_oc_ = (*kT*/*q*) ln­(*J*_sc_/*J*_0_), where exponentially rising reverse saturation current yields ∼2 mV/K voltage decline characteristic of perovskite architectures. Short-circuit photocurrent demonstrates exceptional thermal invariance (31.97 → 32.08 mA/cm^2^) spanning 300–500 K, confirming optical absorption resilience and bandgap thermal stability against moderate heating. Fill factor undergoes progressive deterioration (89.17%→81.46%) attributed to thermally enhanced series resistance and transport layer mobility suppression (ZnO, CBTS). Despite efficiency dropping from 31.75% (300 K) to 23.67% (500 K) as recombination and mobility losses intensify beyond 400 K, retention of >25% efficiency through 450 K markedly surpasses organic–inorganic hybrids failing near 370 K. Superior thermal endurance originates from the all-inorganic architecture featuring Rb^+^ cations that stabilize crystal phases, inhibit ionic diffusion, and minimize structural distortions, qualifying RbGeI_3_ cells for demanding high-temperature outdoor photovoltaic deployment.

It should be noted that the thermal stability trends discussed above reflect only electrical degradation captured within the SCAPS-1D drift-diffusion framework and do not account for the chemical degradation of the absorber itself. A well-documented limitation of Ge­(II)-based halide perovskites is the susceptibility of Ge^2+^ to oxidize to the electronically inactive Ge^4+^ state upon exposure to ambient oxygen and moisture, a process driven by the comparatively low oxidation potential of Ge­(II) relative to Sn­(II) and Pb­(II). This oxidation introduces deep-level trap states and self-p-doping that are not represented in the present drift-diffusion model, and it constitutes the principal practical barrier to realizing the simulated efficiency experimentally. Consistent with strategies reported for related Ge- and Sn-based perovskites, this instability can be mitigated through inert-atmosphere processing and encapsulation, incorporation of reducing additives such as SnF_2_ or hydrazinium salts to suppress Ge­(IV) formation, and compact, pinhole-free CBTS/Au capping layers that limit oxygen and moisture ingress to the absorber.

### 3.6 Interface Defect Density Analysis

The interfacial defect density strongly influences the charge recombination rate and carrier extraction efficiency in multilayer PSCs. To evaluate this effect, the defect density (*N*_t_) was independently varied at the RbGeI_3_/CBTS (HTL/perovskite) and ZnO/RbGeI_3_ (ETL/perovskite) interfaces from 1 × 10^10^ to 1 × 10^20^ cm^–2^. Figure 7 presents the obtained photovoltaic metrics, including PCE, FF, *J*_sc_, and *V*_oc_.

**Figure 7:**
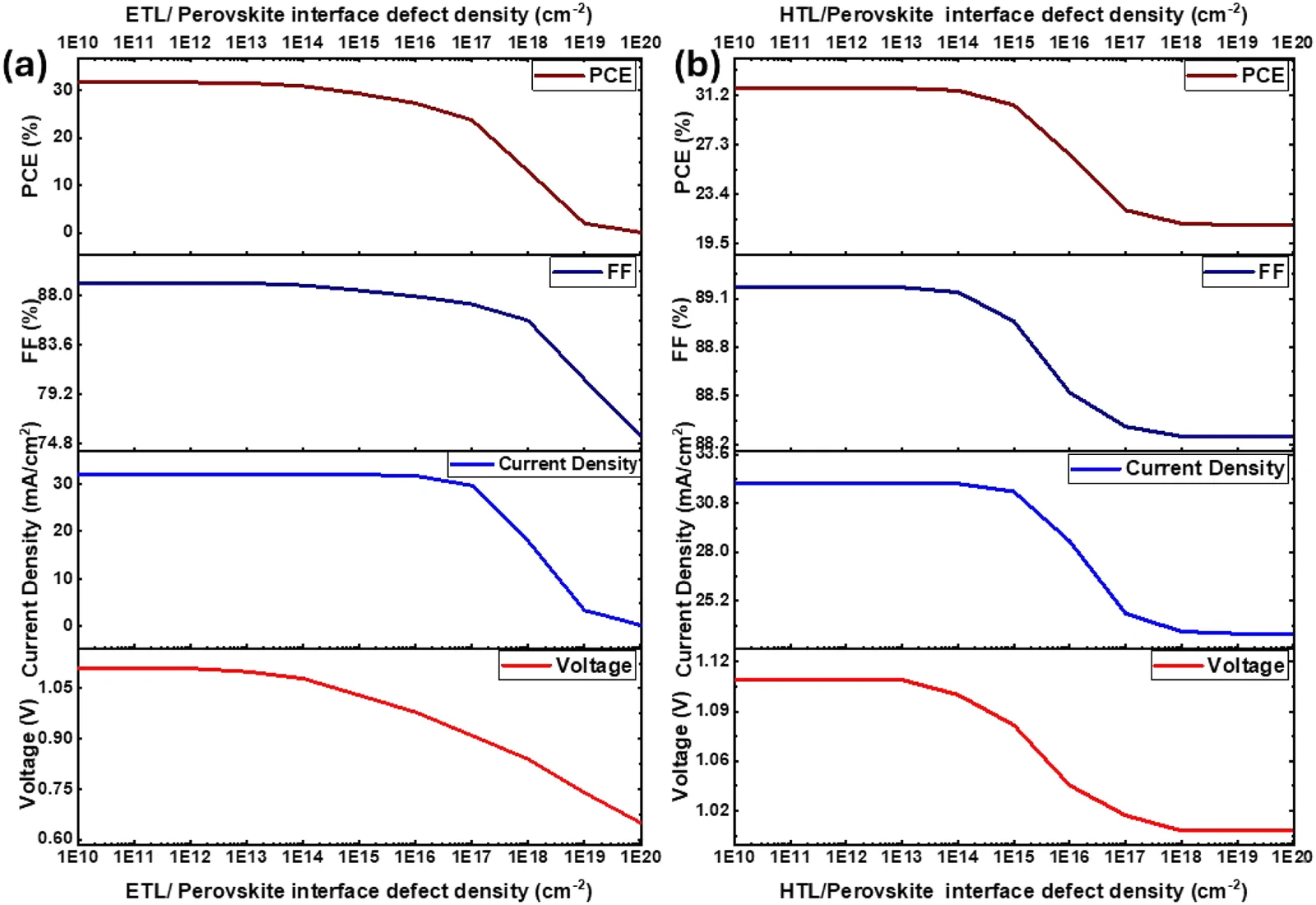
Variation of photovoltaic parameters (*V*_oc_, *J*_sc_, FF, and PCE) with interface defect density for (a) HTL/perovskite and (b) ETL/perovskite junctions, showing the critical role of interfacial passivation in sustaining high device performance.

At the RbGeI_3_/CBTS interface, the device exhibited stable performance up to a trap density of 1 × 10^13^ cm^–2^, maintaining FF ≈ 89.17%, *J*_sc_ = 31.97 mA cm^–2^, *V*_oc_ = 1.11 V, and PCE ≈ 31.75%. A minor drop was observed beyond this value, with efficiency decreasing gradually to 31.56% at 1 × 10^14^ cm^–2^ and to ≈ 20.95% at 1 × 10^20^ cm^–2^. This mild degradation pattern suggests that the RbGeI_3_/CBTS interface forms a relatively defect-tolerant junction due to favorable band alignment and balanced carrier mobilities. However, as the defect concentration exceeds 1 × 10^16^ cm^–2^, SRH recombination becomes dominant, causing a gradual decrease in *V*_oc_ and FF. This behavior highlights that even in lead-free perovskites, HTL interface defect management is crucial for sustaining optimal performance and device longevity.

In contrast, the ZnO/RbGeI_3_ interface displayed a stronger dependence on defect density. The device retained its maximum performance (FF = 89.17%, *J*_sc_ = 31.97 mA cm^–2^, *V*_oc_ = 1.11 V, PCE = 31.75%) up to 1 × 10^12^ cm^–2^. Beyond this threshold, efficiency decreased more sharply compared to the HTL interface. Efficiency fell to 29.4% (1 × 10^15^ cm^–2^), then 13.09% (1 × 10^18^ cm^–2^), and virtually vanished at 1 × 10^20^ cm^–2^ (0.16%). Interface traps at the ZnO/RbGeI_3_ junction act as recombination sites, blocking electron extraction and weakening the internal field, causing this severe performance degradation. Figure 7 highlights the elevated defect susceptibility of the ETL/perovskite boundary relative to the HTL interface. Poor band alignment between ZnO and RbGeI_3_ enhances trap-mediated recombination at this junction, while the better-aligned HTL interface tolerates higher defect densities before degradation becomes severe.

The analysis suggests that maintaining interface defect densities below 1 × 10^13^ cm^–2^ at both contacts is vital for achieving efficiencies above 31%. Values lower than 1 × 10^10^ cm^–2^ theoretically push the PCE beyond the Shockley–Queisser limit (>33%), but such conditions are physically unrealistic in practical thin-film devices and were therefore excluded.

The results underline the necessity of interface engineering strategies, such as surface passivation, buffer interlayers, or atomic-layer-deposited (ALD) coatings, to suppress defect formation at both the ZnO/RbGeI_3_ and RbGeI_3_/CBTS boundaries. Optimizing these interfaces ensures balanced carrier extraction, reduced recombination, and enhanced device reproducibility, which are key factors for translating the simulated efficiency into experimental realization.

### 3.7 Photocurrent–Voltage Response and External Quantum Efficiency (QE)

SCAPS-1D simulations evaluated the FTO/ZnO/RbGeI_3_/CBTS/Au cell under AM 1.5G illumination (1000 W/m^2^, 300 K), with results shown in Figure 8. The device achieved PCE = 31.75%, FF = 89.17%, *J*_sc_ = 31.97 mA cm^–2^, and *V*_oc_ = 1.11 V (Figure 8a). The rectangular *J*–*V* curve indicates minimal resistive and recombination losses with balanced charge extraction. The linear short-circuit behavior and sharp open-circuit transition reflect symmetric carrier mobility across both interfaces. Superior performance results from optimized band alignment and low defect densities. ZnO’s conduction band offset enables selective electron transport while blocking holes, and CBTS provides efficient hole extraction through favorable valence band alignment. The hysteresis-free response indicates stable interfacial charge dynamics.

**Figure 8:**
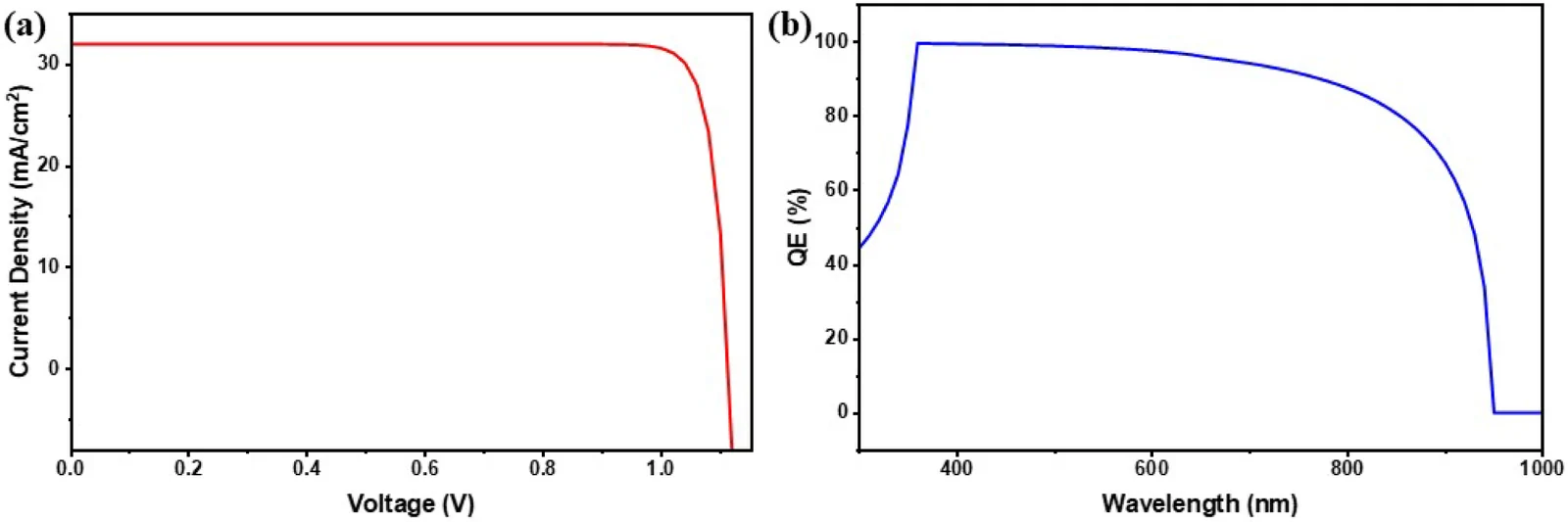
(a) Simulated current density–voltage (*J*–*V*) curve of the FTO/ZnO/RbGeI_3_/CBTS/Au perovskite solar cell. (b) Corresponding external quantum efficiency (QE) spectrum showing ≈100% response in the visible region with an absorption onset near 900 nm.

Figure 8b shows QE approaching 100% between 400 and 750 nm, confirming RbGeI_3_ strong absorption and efficient photoresponse in the visible spectrum. The spectral onset around 900 nm corresponds to the optical bandgap of 1.31 eV, validating efficient sub-bandgap utilization and charge extraction. The slight reduction in QE below 400 nm originates from parasitic absorption losses within the ZnO layer, while the near-infrared decline reflects the intrinsic bandgap limitation of the RbGeI_3_ absorber. The combination of high QE, large *J*_sc_, and minimal recombination loss confirms that the RbGeI_3_ absorber acts as an excellent light harvester in a fully Pb-free system. The optimized architecture balances optical absorption and carrier transport, resulting in a PCE approaching the theoretical efficiency limit for single-junction PSCs.

### 3.8 Comparative Analysis

To assess the importance of the findings from this investigation, the photovoltaic parameters of the optimized FTO/ZnO/RbGeI_3_/CBTS/Au device are compared with recent reports (2023–2025) on Germanium-based PSC simulations. Table 3 summarizes the performance metrics of various architectures utilizing RbGeI_3_ absorbers. The comparative data in Table 3 demonstrates that the proposed ZnO/RbGeI_3_/CBTS architecture achieves a higher PCE of 31.75%, outperforming other contemporary RbGeI_3_ configurations. Specifically, while architectures using SnO_2_/CuI^56^ exhibit a higher *J*_sc_ (44.12 mA cm^–2^), they suffer from significantly lower *V*_oc_ (0.639 V) and fill factor (75.01%), leading to a lower overall PCE.

**Table 3:** Comparative Performance of Recent Pb-Free Ge-Based PSCs

Reference	Absorber layer	Device architecture	PCE (%)	*V*_oc_ (V)	*J*_sc_ (mA cm^–2^)	FF (%)	Year
^51^	RbGeI_3_	TiO_2_/Sb_3_S_3_	30.35	1.06	33.15	85.82	2024
 ^50^	CsGeI_3_	ZnOS/CuSCN	22.89	1.16	23.10	85.35	2025
 ^57^	RbGeI_3_	PCBM/PTAA	21.95	0.83	33.03	79.84	2023
 ^58^	RbGeI_3_	TiO_2_/PTAA	24.03	0.88	33.83	79.85	2025
 ^59^	RbGeI_3_	SnO_2_/CuI	21.15	0.639	44.12	75.01	2025
 ^56^	RbGeI_3_	NZO/GO	23.67	1.05	27.94	80.04	2025
 ^55^	RbGeI_3_	TiO_2_ + 0.5%GrNC/NiO	30.14	1.06	31.98	88.72	2025
This work	RbGeI_3_	ZnO/CBTS	31.75	1.11	31.97	89.17	2026

In contrast, our study reveals that the integration of CBTS and ZnO as the transport layers provides a superior balance of the parameters. The *V*_oc_ of 1.11 V represents one of the peak values for RbGeI_3_ cells, only slightly surpassed by the CsGeI_3_ variant,^50^ while the 89.17% fill factor is the highest reported in Table 3. This high FF indicates excellent charge extraction and minimal interfacial recombination, confirming that the ZnO/RbGeI_3_/CBTS heterojunction provides an optimized band alignment and effective carrier-selective contacts for lead-free photovoltaics with high efficiency.

Both the projected PCE of 31.75% and the FF of 89.17% should be interpreted as SCAPS-1D drift-diffusion limits attainable under ideal Ohmic contacts, defect-free interfaces below the thresholds identified in Sections 3.4 and 3.5, and complete optical generation, rather than as figures expected to be reproduced directly in an as-fabricated device. As Table 3 shows, the highest experimentally adjacent fill factors reported for RbGeI_3_ simulation studies to date cluster around 85–89%, so the present value is at the upper edge of, rather than categorically outside, the range already established for this device family; nonetheless, series and shunt resistance, interface roughness, and nonideal contact behavior typically suppress experimental FF by several percentage points relative to simulation. We further note that, to our knowledge, no experimentally fabricated RbGeI_3_ photovoltaic device has yet been reported in the literature; all entries in Table 3, including this work, are computational studies, so the comparison necessarily remains simulation-to-simulation rather than simulation-to-experiment. We regard the present results as an upper-bound performance target and a set of design rules (absorber thickness, defect-density ceiling, interface quality) intended to guide future experimental realization of RbGeI_3_ devices.

## 4 Conclusion

This integrated computational investigation combining first-principles electronic structure analysis with SCAPS-1D device modeling validates the potential of fully inorganic RbGeI_3_ PSCs, revealing a projected maximum PCE of 31.75% (FF = 89.17%, *J*_sc_ = 31.97 mA cm^–2^, *V*_oc_ = 1.11 V) under optimized conditions. This record-breaking simulated efficiency for a germanium-based system is attributed to the optimal 500 nm absorber thickness and the highly favorable energy band alignment achieved by integrating ZnO and CBTS as transport layers, ensuring highly selective and efficient carrier extraction. Our analysis further established strict material quality requirements: the PCE critically relies on maintaining the absorber bulk defect density below 1 × 10^16^ cm^–3^ to mitigate nonradiative SRH recombination, with the ZnO/RbGeI_3_ junction being the most sensitive interface requiring rigorous passivation strategies. Crucially, the thermal stability analysis demonstrated the inherent robustness of the all-inorganic design, maintaining a substantial PCE of 23.67% even when the operating temperature reaches 500 K. Collectively, these findings position RbGeI_3_ as a highly competitive, environmentally sound, and thermally stable candidate for high-performance photovoltaics, providing precise guidelines for the future experimental realization of next-generation, lead-free solar cells.
